# Detection and Inspection of Steel Bars in Reinforced Concrete Structures Using Active Infrared Thermography with Microwave Excitation and Eddy Current Sensors

**DOI:** 10.3390/s16020234

**Published:** 2016-02-16

**Authors:** Barbara Szymanik, Paweł Karol Frankowski, Tomasz Chady, Cyril Robinson Azariah John Chelliah

**Affiliations:** 1 Szczecin Department of Electrical and Computer Engineering, West Pomeranian University of Technology, ul. Sikorskigo 37, Szczecin 70-313, Poland; 2 Department of Nanosciences and Technology, School of Science and Humanities, Karunya University, Coimbatore, Tamilnadu 641114, India; cyrilraj@live.com

**Keywords:** infrared thermography, microwave heating, eddy current testing, multi frequency eddy current technique, concrete testing, rebar detection

## Abstract

The purpose of this paper is to present a multi-sensor approach to the detection and inspection of steel bars in reinforced concrete structures. In connection with our past experience related to non-destructive testing of different materials, we propose using two potentially effective methods: active infrared thermography with microwave excitation and the eddy current technique. In this article active infrared thermography with microwave excitation is analyzed both by numerical modeling and experiments. This method, based on thermal imaging, due to its characteriatics should be considered as a preliminary method for the assessment of relatively shallowly located steel bar reinforcements. The eddy current technique, on the other hand, allows for more detailed evaluation and detection of deeply located rebars. In this paper a series of measurement results, together with the initial identification of certain features of steel reinforcement bars will be presented.

## 1. Introduction

Reinforced concrete has been an universally dominant construction material for over a century, although structures made of this material are often exposed to many types of damage and deterioration due to different causes and exposure conditions. Therefore, in most countries relatively frequent inspections are required due to building code requirements. Usually, such tests should be conducted after the concrete has hardened and without damaging the structure. For this reason nondestructive testing methods (NDTs) are commonly used for this evaluation [1,2,3]. There are several aspects in reinforced concrete testing, which are important from the practical point of view:

- Assessing the dimensions of structural elements and locating damage and defects (such as voids, cracks and inclusions). Here the most popular NDT methods are: ultrasonic sensors and ground penetrating radar [4,5,6,7,8,9,10,11,12]. Both methods are fast and reliable, but the results obtained are not easy to interpret. Active and passive thermography can also be used to inspect the inner structure of concrete, but due to some limitations, in practice these techniques are used to detect defects or plaster damages near the surface [12,13].
- Reinforcement location and corrosion assessment. Here the natural choices are electro- magnetic methods, such as radiography (a very efficient method, but hard to use in practice and dangerous for the operator) [14,15,16,17], eddy current sensors (a promising method, that allows not only the detection of the reinforcement, but also identification) [18,19,20] and impedance tomography, which is, in contrast to previously mentioned methods, a contact technique.
- Dampness assessment. Here the most commonly used methods are impedance tomography [21,22,23], thermography and some chemical techniques.

In this article the authors propose a new concept of two-step concrete structures analysis, using two different non destructive techniques: active thermography and eddy current sensors. The active thermography method is proposed to be used as a preliminary technique, which may be useful in initial rebar detection, whereas the eddy current technique is used for structure assessment and damage identification.

In the case of active thermography, an external energy source should be used to induce a thermal contrast inside the inspected specimen. In the case of concrete structures conventional heating methods (like halogen lamps, flash lamps, infrared radiators, hot air and so on) may usually be not effective due to the large size of the inspected structures. In this study we propose using microwave heating [24,25,26,27]. In the literature microwave heating of concrete structures is used mainly for acceleration of curing, cement decontamination and drilling or melting the concrete [28]. Here we propose the microwaves as the energy source in infrared thermography (this concept is very rare in the literature, and the authors are familiar with only one publication concerning this subject [24]). The main advantage of the microwave heating is its volumetric character. A certain volume of the specimen is heated at once, which means that this technique is very fast. On the other hand, in the case of this non-conventional energy source, the heating ratio is dependent not only on the thermal properties of the material, but also on some electrical properties. Also an interesting phenomenon is observed during the interaction of the microwaves with metal—The heating is strongly limited due to a very low penetration depth value. This phenomenon will be demonstrated in both numerical models and experiments. To the best of the authors' knowledge the numerical modeling of reinforcement detection in concrete structures using microwave heating has not been previously described in the literature. After heating, a thermovision camera is used to observe the inspected specimen surface. The inner material flaws and inclusions will cause the non-uniform heating of the sample, and this will be visible as hotter or cooler places. Therefore steel bars should be easily detected using this method. Due to the large scale of the inspected structures and the characteristics of concrete heating (which will be further discussed), this method will be here used as a preliminary study technique.

The second method proposed here, the multi-frequency eddy current technique, may be used as a second inspection step as it allows not only for the detection of the rebars, but also identification of some features of these structures. Previous researches prove that the single-frequency eddy current method (SFM) can be successfully used to evaluate many structural parameters. The identification may involve: rebar diameter (*D*), physical properties of rebar (represented by rebar class), and rebar location, including thickness of the concrete cover (*h*). Therefore, the eddy current evaluation is one of the most promising NDT methods. Nevertheless, the same tests prove that evaluating three parameters at once is a problem, especially, when the concrete cover thickness is high.

In this paper, the aim is to present the multi-frequency method (MMFM) which can be used as an alternative to the standard assessment [29,30,31,32]. However, MMFM can also be utilized complementarily to the SFM. During the investigation, it is very important to correctly select the frequency and transducer size, especially the distance between the transducer columns. Small transducers have good spatial resolution, while bigger transducers are more sensitive. Therefore, small transducers work correctly only when distance between the transducer and a ferromagnetic material is low, so in this case the frequency used should be high. The larger transducers provide better results greater distances, however their spatial resolution and repeatability are lower. The MMFM allow testing over 50 different frequencies during the one measurement and easily selecting the best one. Also, if transducer size is too small or too large for a particular sample, the test will show this up. In this paper we present the new method of frequency selection in MMFM, and we also present suitable statistical data mining methods that are used to obtain signals in the multi-frequency method allowing for very efficient structure identification.

## 2. Active Infrared Thermography with Microwave Excitation

The mechanisms of microwave heating of a variety of materials are well known and have been extensively described in the literature [4,9]. Obviously, this heating is very closely correlated with the incident electric field frequency—for lower microwave frequencies the ionic conductivity is the most important cause of the material heating, whereas for higher frequencies the energy absorption is primarily due to effects connected with the polarity of the materials' molecules. In our study the incident microwave frequency is fixed at a 2.45 GHz value—The most common frequency for industrial and domestic purposes. As mentioned previously, in this case the heating mechanism is primarily due to the existence of dipole molecules which tend to re-orientate in the presence of a microwave field. The loss mechanism is due to inability of the polarization to follow the fast field alternations.

The rate of microwave heating is dependent not only on the thermal properties of the material, but also on some of its electric properties. An important factor is the value of the dielectric loss tangent tanδ = ε''/ε' where *ε*’ indicates the real part of complex dielectric permittivity, and ε'' is its imaginary part. The dielectric loss tangent is a function of the frequency, and describes the ability of a material to absorb the microwave energy: the higher its value, the more energy can be absorbed by the material.

In case of metals, and other materials with very large electric conductivity values, the crucial factor in case of microwave heating is penetration depth, described by:

(1) $$\delta =\sqrt{\frac{1}{\pi \mu \sigma f}}$$

where *f* is the electric field frequency, *μ* is the magnetic permeability of the material and *σ* is the material conductivity. In our case, for steel rebar the penetration depth is equal to about 4 μm, which in practice may be treated as negligible. This basically means that metal (even if not treated as a perfect conductor, with resistivity equal to zero) can be considered as a microwave-reflecting material.

### 2.1. Numerical Modelling of Microwave Heating

The numerical modelling was performed using commercial software-COMSOL Multiphysics ver. 4.4 (COMSOL, Inc., Burlington, MA, USA). This software is a powerful tool for solving partial differential equations using Finite Element Method (FEM). The main advantage of this tool relates to its ability to solve multiphysics problems—*i.e.*, ones where several physical phenomena are connected. Microwave heating is an example of a problem where one has to take into account more than one physical module.

**Figure 1:**
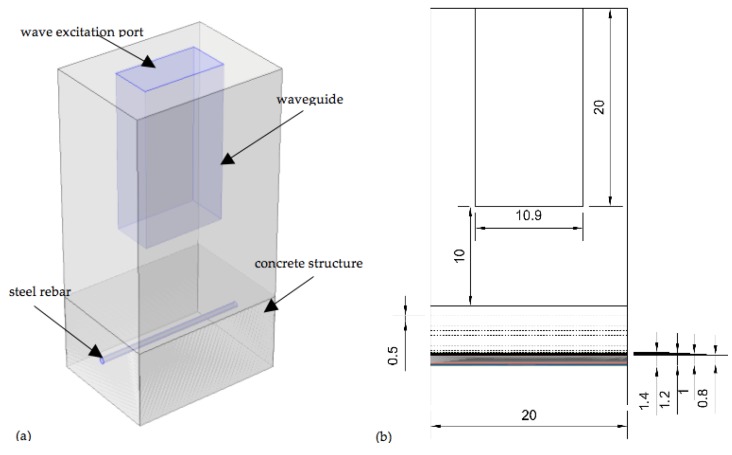
(**a**) The geometry used for 3D numerical modelling in COMSOL; (**b**) problem parameterization: the chosen diameters of steel rebar and its position below the concrete surface are shown.

The created model was based on several governing equations—Electromagnetic wave equation, electromagnetic power dissipation equation derived from Poynting theorem and the heat transfer equation. Therefore two COMSOL modules were used: “Electromagnetic waves”, solved in the frequency domain and “Heat transfer in solids”, solved in the time domain. These modules were coupled by using the domain condition “Heat source” defined as the density of total electromagnetic power dissipated in the material. Figure 1a presents the model's geometry. The position and diameter of the rebar was parameterized as shown in Figure 1b. Four rebar dimension values were analysed (0.8, 1, 1.2 and 1.4 cm) and nine rebar positions below the concrete surface (from 5 cm to 1 cm in 0.5 cm steps).

The wave excitation was carried out using the COMSOL port boundary condition, with the power fixed at 1000 W. The time of heating was set to 60 s, and after that the natural cooling stage of the system was simulated. Total time of simulation was set to 360 s. The simulated material properties of concrete, air and steel are collected in Table 1.

As previously mentioned, the simulation was parameterized in terms of the rebars' diameter and the position of the rebar below the concrete surface. As a result we performed 36 simulations, one for each case.

**Figure 2:**
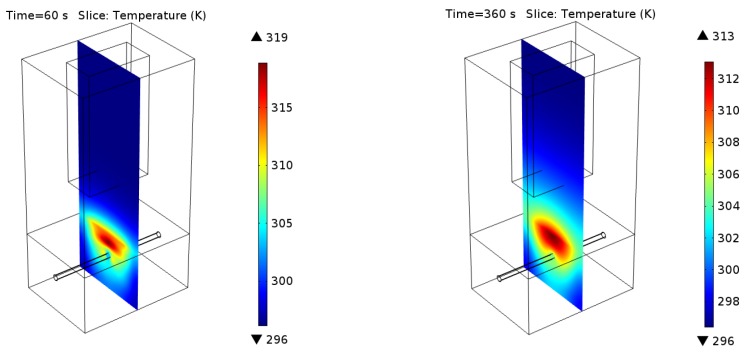
Exemplary results of numerical modeling (rebar diameter = 10 mm and position = 40 mm below the concrete surface). The comparison between the temperature distributions after the heating phase (60 s) and cooling phase (360 s) is shown.

**Figure 3:**
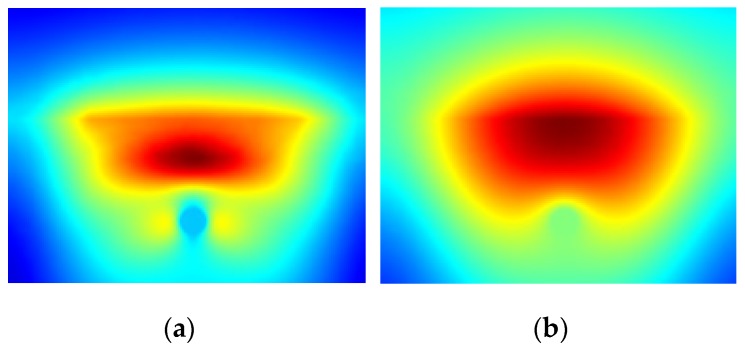
Zoomed region of interest. The comparison between the temperature distributions after: (**a**) the heating phase (60 s) and (**b**) cooling phase (360 s).

To present an exemplary result, the temperature distribution was plotted for one slice, located in the middle of the geometry, in the *yz* plane (see Figure 2). The temperature was plotted for the chosen rebar's diameter and position (*i.e.*, diameter = 10 mm and position = 40 mm below the concrete surface). The first plot (Figure 2a and Figure 3a) shows the temperature distribution after the heating process (the first 60 s of the simulation). It is clearly visible that the steel rebar is not heated by microwaves. Heating itself is not uniform, the heated volume is dependent on the waveguide flange shape (here we simply use an open waveguide) and the distance between the waveguide and the sample (here, 10 cm). Obviously the temperature distribution is also disturbed by the presence of the steel rebar inside the concrete structure. It is clear that the resulting images should be properly analyzed to obtain an image of the detected rebar.

**Figure 4:**
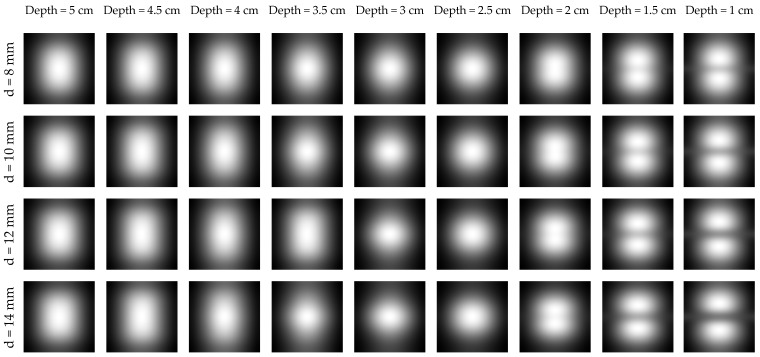
The temperature distribution at the concrete surface obtained for all rebar diameters (*d*) and depths.

It can be clearly seen in Figure 2 and Figure 3, that in most cases detection of the rebar can not be done directly (based on the raw data). Although the rebar itself is not heated and the temperature distribution, observed at the concrete surface, has to be disturbed by its presence, the effect is hidden because the concrete itself is heated quite effectively by microwaves too. The raw data for an exemplary time step (200 s) for all configurations is shown in Figure 4.

**Figure 5:**
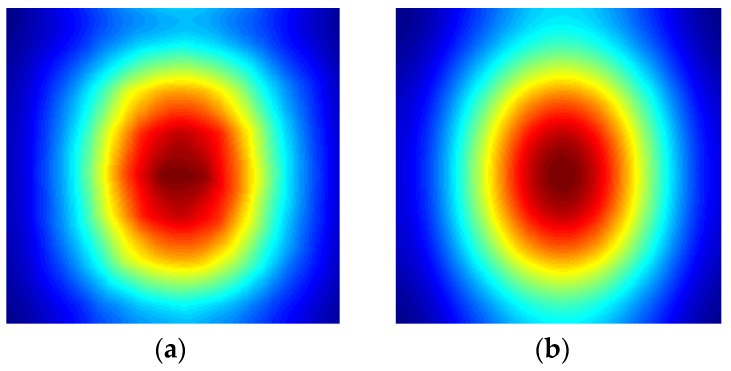
The temperature distribution at the concrete surface obtained in a model without the rebar. (**a**) observed after the heating phase (60 s), (**b**) after the cooling phase (360 s)

**Figure 6:**
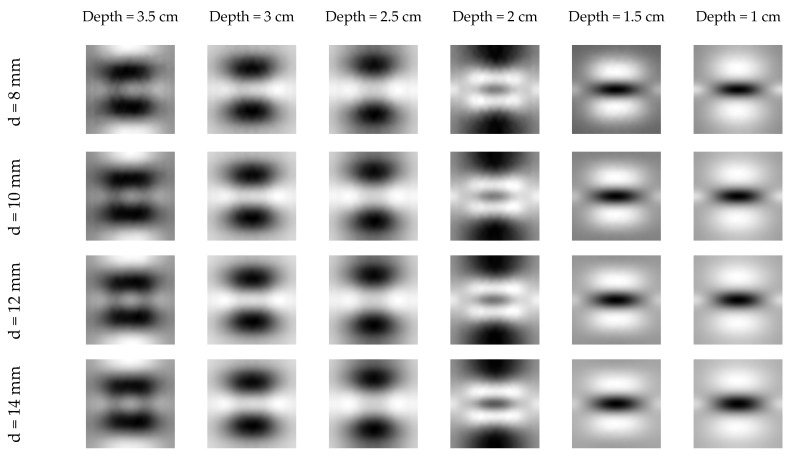
The result of subtracting the temperature profile obtained in a model without the rebar from the original data. Results for all the rebar diameters and chosen depths.

It is clearly visible that for all rebar diameter cases the object can be detected only if it is located shallowly beneath the concrete surface (*i.e.*, 1 cm, 1.5 cm and 2 cm). In case of numerical modeling it is possible to model the case with solid concrete structure, without the steel bar inside (in Figure 5 one may see the temperature distribution at the concrete surface for this model), which may be used to remove the effect of concrete heating, by simple subtraction. The results of such operation for an exemplary time step (200 s) are shown in Figure 6. The results are not easy to interpret, as the rebar is once indicated as a hotter (lighter gray) place and once as a cooler (darker gray) spot in the middle of the image. This phenomena may be explained after the insightful analysis of the heating/cooling process of the modeled system. When the rebar is located deeper beneath the concrete surface it limits the volume heated by the microwaves. In such a situation, the temperature just above the rebar will be much higher, than the temperature at the same spot in the model without the rebar. The same phenomenon occurs, obviously, in the case when the rebar is located shallowly, but the cooling process for small volumes is fast enough, so the rebar is visible as the cooler spot.

**Figure 7:**
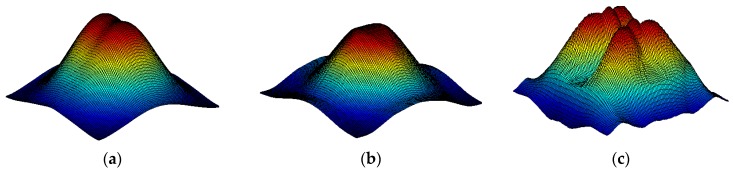
Visualization of the background removal method based on median filtering, shown for the case of rebar with 14 mm diameter located at 2 cm depth. (**a**) original data, (**b**) data after median filtering with large mask, (**c**) results of subtraction (**a**,**b**).

**Figure 8:**
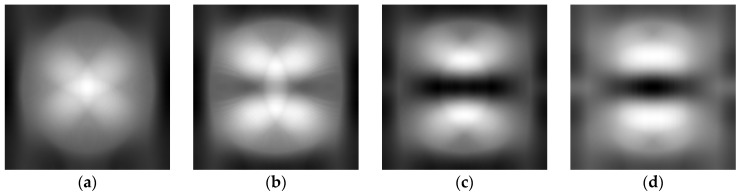
The result of background removal for the chosen case. Rebar diameter—14 mm, (**a**) depth—2.5 cm, (**b**) depth—2 cm, (**c**) depth—1.5 cm, (**d**) depth—1 cm.

Obviously, in reality, one does not have the access to the case of solid concrete, so the previously shown operation has to be replaced by some numerical estimation of the trend. The idea is to use median filtering with a large mask. Finally, the obtained data is subtracted from the original image. This is one of the methods used in background removal from the thermal images [33]. This process is shown in Figure 7. In Figure 8 one can see the result of such an analysis for one case of rebar diameter (14 mm, for the remaining cases, the results are similar). It can be clearly seen that the rebar detection is limited and possible only in the case when it is located shallowly beneath the concrete surface (*i.e.*, 1 cm, 1.5 cm and 2 cm).

### 2.2. Experimental Methods and Results

The numerical modeling has shown that the detection of rebar is possible using active thermography with microwave excitation. To validate the previous results, a series of experiments have also been conducted.

The experimental setup is composed of a microwave heating device, an A325 thermovision camera (FLIR Systems, Inc., Wilsonville, OR, USA) and the tested sample. A magnetron with an output power of 1 kW, operating on 2.45 GHz, connected directly to rectangular waveguide, is used as a high power microwave generator. The open waveguide's aperture is pointed directly at the sample surface. For safety purposes and experimental accuracy the experimental setup is located in an anechoic chamber.

**Figure 9:**
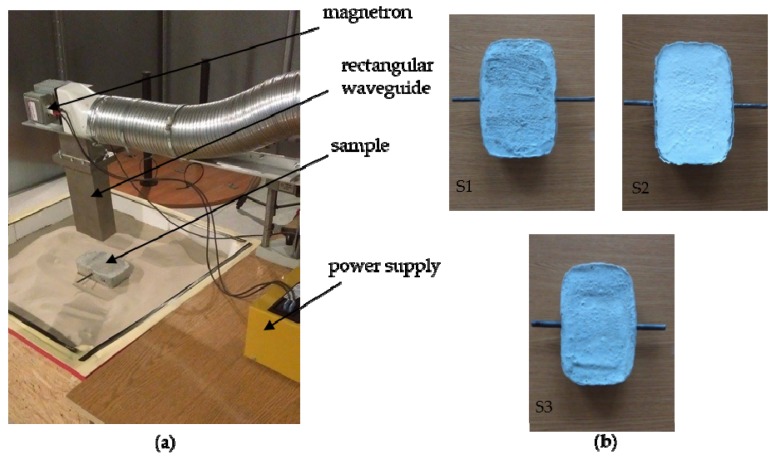
(**a**) photo of the experimental setup, (**b**) samples: S1—rod diameter 8 mm, S2—rod diameter 10 mm, and S3—rod diameter 12 mm.

**Figure 10:**
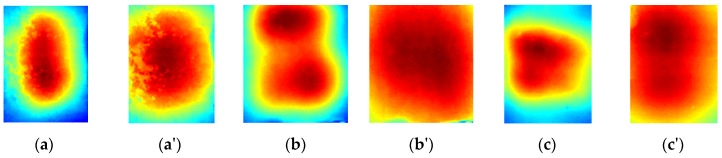
Thermograms obtained for each sample: S1 (**a**) after the heating phase, (**a'**) after the cooling phase, S2 (**b**) after the heating phase, (**b'**) after the cooling phase, S3 (**c**) after the heating phase, (**c'**) after the cooling phase.

Figure 9 presents the described setup. Three samples, with different steel rebar diameters (8, 10 and 12 mm) were tested. The position of the rod was set to 1.5 cm below the concrete surface. The time of heating was set to 60 s. For safety reasons, the sample was not observed during the heating phase. After the heating the sample's surface was observed using a thermovision camera for an additional 5 min, during the natural cooling process. In total, for each sample sequences of 300 thermograms were recorded (1 thermogram per second). Figure 10 presents the raw thermograms acquired for each sample, just after the heating (1st thermogram in the sequence) and at the end of cooling process (300th thermogram from the sequence). It can be noticed that the results obtained experimentally are quite similar to those from numerical modeling.

**Figure 11:**
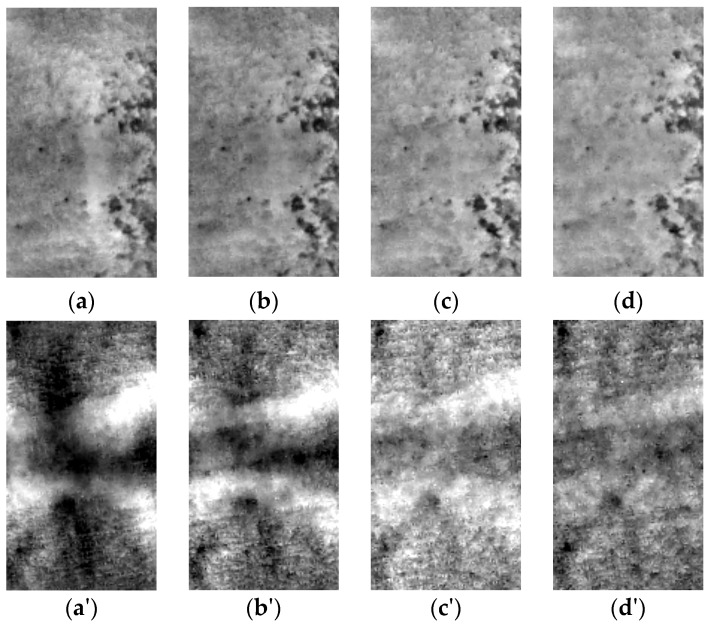

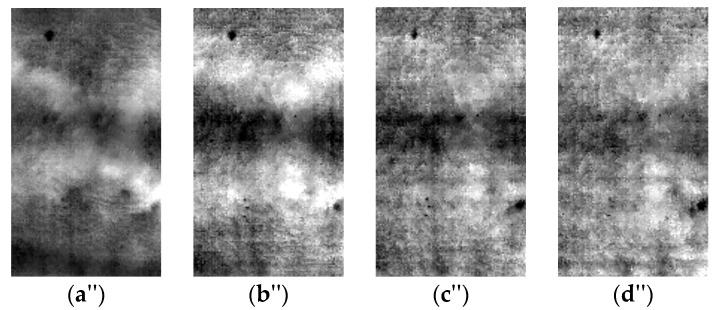
The results of background removal obtained for all samples. S1 (**a**) 1st thermogram in the sequence, (**b**) after 100 s, (**c**) after 200 s, (**d**) after 300 s, S2 (**a'**) 1st thermogram in the sequence, (**b'**) after 100 s, (**c'**) after 200 s, (**d'**) after 300 s, S3 (**a''**) 1st thermogram in the sequence, (**b''**) after 100 s, (**c''**) after 200 s, (**d''**) after 300 s.

In the case of the experimental data the previously shown technique of background removal was also used. The results are shown in Figure 11. It can be noticed that this simple image processing technique enables detection of the rebars in the case of sample S3. Also for sample S2 the rebar position is visible. It can also be noticed, that the best result can be obtained after 100 s of observation. For sample S1 the rebar detection was not successful. This result shows the method limitations, related not only with rebar depth (which was shown in numerical modeling), but also its diameter.

## 3. Eddy Current Technique

The second technique discussed here may be used both to detect and evaluate rebars. The eddy current method uses the phenomena of electromagnetic induction to detect inner flaws or inclusions in the examined materials. This method is a natural choice in the case of conducting materials and the possibility of using it in rebar detection has been discussed earlier [34,35,36,37]. Here we present the multi-frequency technique and we propose a method that may be used for the problem of identification of chosen parameters of the rebar on the basis of obtained signals. This method should be considered as complementary to thermovision. The combination of both methods may create the full, multisensor system for rebar detection and identification.

### 3.1. Single Frequency Methods

**Figure 12:**
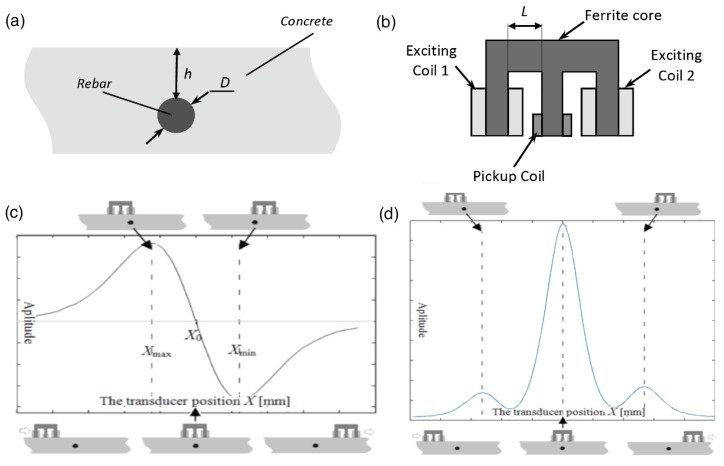
(**a**) Tested specimen; (**b**) Cross-section of the transducer; (**c**) Example of the signal received at the pick-up coil of the differential transducer; (**d**) Example of the signal received at the pick-up coil of the absolute transducer.

**Figure 13:**
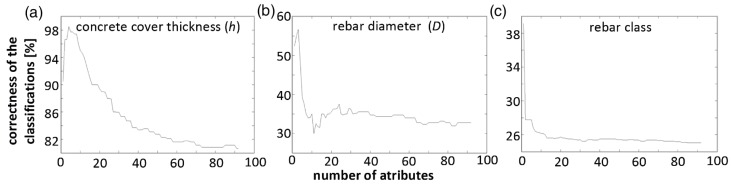
Simple classification model correctness of classification *vs.* number of attributes. Parameters: (**a**) concrete cover thickness, (**b**) rebar diameter, (**c**) rebar class.

The system utilized was presented in previous work [34,35]. It consists of an E-shaped differential transducer (Figure 12b) and four subsystems: an XYZ scanner, an excitation subsystem, a data acquisition subsystem and a controlling computer. The exciting coils of the eddy current transducer are powered by two independent function synthesizers through power amplifiers. The frequencies of the both excitation sinusoidal signals are equal. The alternating currents in the two excitation coils have the same amplitude and opposite phase in the case of differential mode or the same phase in the case of absolute mode. In the case of the differential transducer, excitation coils create equal but opposite directed magnetic fluxes that cancel each other out. Therefore, in equilibrium state a voltage measured at the pick-up coil is small and constant. Variations of the electrical conductivity or magnetic permeability of the tested object, or the presence of flaws, cause changes in the eddy current flow and corresponding changes in the phase and amplitude of the measured voltage. An example of the signal achieved during movement of the transducer above the specimen with a single rebar is shown in Figure 12c,d. The measurements were taken in steps of 0.61 mm for different thicknesses of the concrete covers and different samples (the rebars having different diameters *D* = {8,10,12,14} mm, and belonging to three classes). For the SFM two kinds of attributes are used in the analysis: the shape factors *d* and the maximal value of the waveform *U*_max_. Values of the shape factors *d* are independent of the waveform maximal value and describe only the shape of a normalized waveform (e.g., *d*_30_ is a difference between *X*_max_ and *X*_30_, where X_30_ represents position of the transducer when the signal amplitude at the pick-up coil was equal *U*_30_ = 30%·*U*_max_). At the beginning almost 100 attributes were considered. However, in classification tasks when the problem dimensionality increases, the volume of the space increases very fast and the available data become sparse. In order to obtain a reliable result, the amount of data needed to support the result often grows exponentially with the dimensionality. The curse of dimensionality can be avoided by reducing the number of dimensions. Figure 13 presents this phenomena achieved in the case of results obtained for very simple model including Kullback–Leibler divergence attribute filter and Naive Bayes classifier.

The simple model presented can be used to determine the complexity for every particular classification task (Figure 13). However, the results obtained are not satisfactory. A final model was built based on the rough set theory [7,9]. This method allows one to presents relations between received voltage waveform and tested sample in the form of “if/then” rules. Moreover, the method also allows the selection of an optimal attributes set. During the tests a relatively large transducer was used (T_20_). As one can see in Table 2, the transducer achieves its best performance for a concrete cover thickness range *h* from 15 to 35 mm. For these distances not only is the classification correctness the highest, but also the attribute standard deviation is the lowest (statistics were calculated based on the database including 110 instances for every value of the concrete cover thickness *h*). Studies showed the importance of the correctly selected transducer size, especially the distance *m* between the transducer columns (between the pick-up coil and the excitation coil). In this research three different transducers were used: T_25_ where *m* = 25 mm, T_20_ where *m* = 20 mm and T_5_ where *m* = 5 mm. The smaller transducers have good spatial resolution, while bigger transducers are more sensitive. This means that the small transducers work correctly only when the thickness of the concrete cover (*i.e.,* the distance between the ferromagnetic bar and the transducer) is small. The larger transducer provides better results for a bigger thickness *h*, however its spatial resolution and repeatability are low. Moreover, large size transducers provide relatively good results for a big interval of *h* values. Small transducers should be used when the distance between the pick-up coil and the sample is small and when the interval of distances *h* is small. Results obtained for the three different transducers are presented in Table 3. Both kinds of the transducer operation modes were evaluated. In many cases the differential eddy current transducers are more effective. Small magnitudes of voltage on the pick-up coil result in very high sensitivity, therefore these kinds of transducers are perfect for crack detection and evaluation. In this case, the quality of results collected by the SFM for both types of connection is comparable. However the MMFM is more effective when the transducer coils are connected in other ways. Therefore, results presented in the next section were collected using absolute connection.

### 3.2. Massive Multi-Frequency and Spectrogram Method

**Figure 14:**
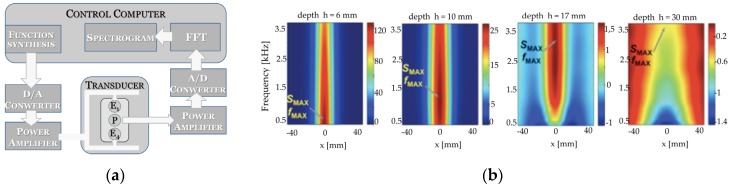
The Massive Multi-Frequency Method (**a**) system; (**b**) basic concept of identification.

The MMFM [18,19] is based on a few simple physical phenomena. The first one is an eddy current phenomenon. The other is the fact that low frequencies have a large skin depth and hence give clear signals from support structures that are located away from the coil. Because of the different depths of penetration for different frequencies, the relationship between signals for different samples (*h*) changes with frequency. Consequently, it is possible to combine the signals from many different frequencies. It was proved [29,30,31,32] that the MMFM system allows changes of the reinforcement structure to be observed through changes in the corresponding spectrograms. The frequencies for which the amplitude of components display the largest changes depend on changes in the tested structure [32]. This can be used not only for measuring the thickness of conductive coatings on conductive base metals, detection and identification of flaws in conducting materials, or differentiating between flaws in various layers of built-up structures, but also in the task of evaluating the distance between a transducer and ferromagnetic elements [29,30] or identification of ferromagnetic shapes or material. An additional advantage of the utilization of numerous frequency components in the excitation is that every single frequency consists of independent information, therefore, the impact of noise can be reduced. Moreover the presented method is much faster than Swept Frequency Methods. The scheme of the system [31] is presented in Figure 14a. Exciting coils are excited simultaneously by the multi-frequency signals given as:

$$y\left(t\right)=\sum_{i=1}^{n}U_{i}\cdot \sin\left(\varpi_{i}\cdot t+\varphi_{i}\right)$$

**Figure 15:**
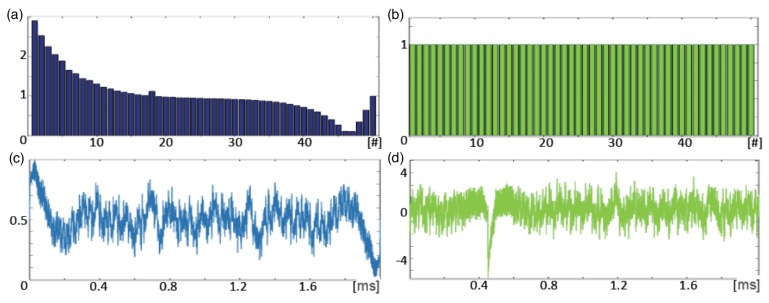
The frequency spectral of signal magnitude fed to: (**a**) exciting coils; (**b**) pick-up coil. The one period of time signal fed to: (**c**) exciting coils; (**d**) pick-up coil.

The transducer is moved over the sample. The voltage waveform measured during the movement at the pick-up coil is decomposed by a FFT algorithm. Normally, when a sample is uniform, the FFT products can easily be set equal. However, to receive equal output signals the magnitude of the excitation signal components must be precisely selected, as presented in Figure 15. In order to build the multi-frequency excitation signal 45 components having frequencies from 0.5 kHz to 200 kHz were used (Figure 15). The frequency set was selected with inconstant steps, that is, the size of the steps changed logarithmically to show the phenomena properly. The basic concept of MMFM is based on the spectrogram peak [29] (highest value in spectrogram) parameters (*S*_max_, *f*_max_) (Figure 14b). When the distance between the transducer and rebar becomes larger, then the spectrogram peak *f*_max_ is shifted towards higher frequencies. An example of a frequency spectrum of typical input and output signals is presented in Figure 15.

### 3.3. Results and Identification

The MMFM can be used to select the best frequency for SFM inspection [19]. From the investigator’s point of view the most interesting is the frequency where changes in the received signal are the largest. Therefore, in most cases the best testing frequency is *f*_max_, the frequency where the peak value *S_max_* is observed.

**Figure 16:**
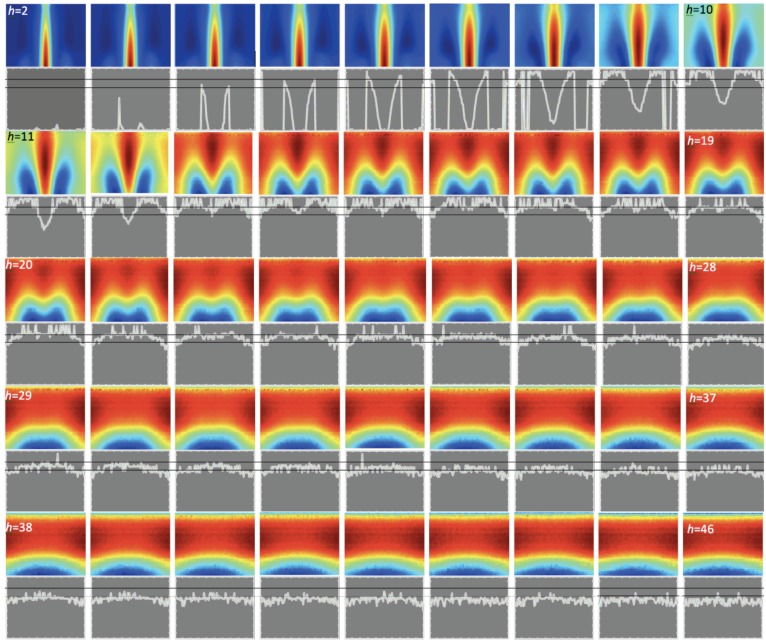
Spectrograms and spectrograms max. value profiles created for 45 different values of *h* .

The method can also be used to select the best among available transducers. Table 2 and Table 3 show that each transducer has a range of distances from particular sample, for which it works properly. In the SFM measurements to detect this range interval it was necessary to build the corresponding database and calculate the statistics. During the tests 110 measurements for each *h* were carried out. A much faster way to get the same result is to use the MMFM and create the corresponding spectrograms (Figure 16). The largest changes are observed for the spectrograms achieved for *h* values between 7 mm to 30 mm. The differences between spectrograms obtained for intervals of *h* between 2 mm to 7 mm and 30 mm to 40 mm are also significant. For higher values of concrete cover thickness the changes are small, therefore identification can be difficult. The statistical results presented in Table 2 and Table 3 could be expanded to a larger depth interval by increasing the number of excitation frequencies. Therefore large transducers can be utilized also for low distances. This is one of the biggest advantages MMFM has over the SFM, because one large transducer can be used over the whole range.

The basic identification process is based on the *f*_max_. However, studies show that the changes of *f*_max_ are observed only for the 5–15 mm range. The identification can be conducted for much wider intervals of *h*. To make this possible, profiles of maximal value spectrograms are used. These profiles show local *f*_max_ values (a component with the highest magnitude) for every single transducer position (Figure 16, below the spectrograms).

During this investigations the assessment of distance between the ferromagnetic material and transducer was considered the simplest task (Figure 13). However like with SFM, the set of most useful attributes was selected and association rules were inferred [35,36]. Rough set theory was used for this purpose [38,39].

For low *h* identification can be carried out based only on three parameters: global *f*_max_, and two local *f*_max_. The first one is a local *f*_max_ for transducer position when one of the excitation coils is directly above the rebar. This attribute is very significant when the distance between transducer and rebar is small. The other one is a local *f*_max_ taken when the rebar is placed over 40 mm from the pick-up coil (when the impact of the eddy current is small). This one is significant when the distance between transducer and rebar is higher than 30 mm. Based only on these three parameters it is possible to get about 95% correct identifications (if we know the rebar diameter and class). However, the most universal and useful attribute is the surface area under the profile line. This attribute allows one to expand very effectively the identification over the whole range. Even if the other parameters of the structure are unknown, the classification error is still very small and differences between real *h* and predicted *h* are smaller than 2 mm.

The identification of the rebar diameter is even more difficult. Attributes from the previous case are the most significant. However to improve the correctness of classification it is necessary to add a few more attributes. The number of attributes and correctness of classification in this case depends on transducer size and the method used for identification of a rebar class and ranges between 92%–98%. The last case is rebar class identification.

The class classification is relatively difficult when conducted based only on the profile surface area, and local/global *f*_max_ parameters. However, correctness of classification still can be around 90%. Previous investigations have shown that the rebar class is strongly correlated with the signal magnitude [34]. Therefore, to get better results it is reasonable to add *S*_max_ to the database or the maximal value of all frequency waveforms.

## 4. Conclusions

Thermography with microwave excitation allows for the rough detection of rebar reinforcements. This method can be used on large areas (using for example antenna arrays) which may simultaneously be observed using a thermovision camera. This is the main advantage of this technique. According to the measurement and numerical simulation results, changes in the rebars’ diameter (in the range between 8 and 14 mm) has a very moderate influence on their detectability. Unfortunately the method has also some strong limitations. Detection of rebars becomes difficult when the rebars are located deeper than 20 mm under the surface of the concrete. This issue can be overcome by utilizing signal processing techniques like principal component thermography. Another solution is to use an additional different technique. In this paper the authors proposed the successful multi-frequency eddy current method as a complementary diagnostic tool.

The massive multi-frequency method (MMFM) allows for fast, easy and accurate identification of the reinforced concrete structure parameters. Although the MMFM requires building more complex hardware and software than the SFM system, it has many advantages. Large numbers of frequency components in the excitation signal allow the full testing of a structure with high accuracy, a far bigger than it is possible with SFM. The method can also be used to expand the operating range of the transducer and the identification process is easier than in the case of the single frequency method. The method can therefore be used as a substitute or a complementary technique to the standard single-frequency identification method. In summary, the multi-frequency method is more effective, but also more complex than the single-frequency one.

## Figures and Tables

**Table 1:** Basic thermal and dielectric (at 2.45 GHz) properties of materials used in simulations.

Material	Loss Tangent tan *δ* = *ε*'' / *ε*'	Density [kg/m^3^]	Thermal Conductivity [W/(m·K)]	Heat Capacity at Constant Pressure [J/(kg·K)]
Concrete	0.36/4.5	2400	0.8	750
Steel	Here simulated as conductivity σ = 8.41 × 10^6^ S/m	7850	66	490
Air	-	1.29	0.022	1010

**Table 2:** Correctness of the classification and standard deviation of selected attributes.

*h* [mm]	5	10	15	20	25	30	35	40	45	50
D	75%	81%	91%	96%	92%	93%	84%	75%	78%	66%
Class	78%	83%	92%	100%	87%	94%	95%	79%	78%	74%
**σ_d80_**	0.530	0.480	0.295	0.257	0.295	0.295	0.257	0.498	0.561	1.114
**σ_d10_**	5.986	4.259	0.707	0.450	0.450	0.502	0.518	1.068	1.179	1.841

**Table 3:** Transducer parameters and statistics.

Transducer size	T5	T20	T25
Optimal range of *h* [mm]	0–25	15–35	15–35
Correctness of D classification in the optimal range [%]	94–98	84–96	91–93
Correctness of D classification for *h* = 40 to 50 mm [%]	58–68	66–78	72–78
